# Construction and verification of a predictive model for depression risk of patients with somatization symptoms

**DOI:** 10.3389/fpsyt.2025.1555513

**Published:** 2025-04-01

**Authors:** Liming Tang, Jinrong Zhong, Mei’e Zeng, Weiwei Deng, Chunmei Huang, Shuifen Ye, Fengjin Li, Dongqin Lai, Wanling Huang, Bin Chen, Xiaoyuan Deng, Xiaoying Lai, Lirong Wu, Bilan Zou, Hanzhong Qiu, Ying Liao

**Affiliations:** Department of General Medicine, Longyan First Affiliated Hospital of Fujian Medical University, Longyan, China

**Keywords:** predictive model, depression risk, somatization symptoms, clinical 49 validation, risk factors identification

## Abstract

**Background:**

Patients with somatization symptoms are at elevated risk of depression, yet underdiagnosis persists due to cultural tendencies (e.g., in China) to express psychological distress via physical complaints. Existing predictive models lack integration of sociocultural and physiological factors, particularly in non-Western populations.

**Objective:**

To develop a culturally tailored risk-prediction model for depression in patients with somatization symptoms, emphasizing early identification and personalized intervention.

**Methods:**

A prospective cohort study included 200 somatization patients (SSS≥38, PHQ-2<3) from a Chinese hospital (May 2020–August 2022). LASSO regression identified predictors from 18 variables, followed by multivariate logistic regression to construct a nomogram. Model performance was assessed via ROC-AUC, calibration curves, Hosmer-Lemeshow test, and decision curve analysis (DCA). Internal validation used 200 bootstrap resamples.

**Results:**

Five independent predictors were identified: advanced age (OR=1.11, 95% CI: 1.02–1.20), poor self-rated health (OR=2.07, 95% CI: 1.04–4.30), lack of co-residence with children (OR=1.63, 95% CI: 1.10–2.42), low income (OR=1.45, 95% CI: 1.05–2.01), and self-medication (OR=1.32, 95% CI: 1.01–1.73). The nomogram demonstrated strong discrimination (AUC=0.810, 95% CI: 0.728–0.893) and calibration (Hosmer-Lemeshow p=0.32). DCA confirmed clinical utility: at threshold probabilities >5%, the model provided higher net benefit than “treat-all” or “treat-none” strategies.

**Conclusion:**

This model integrates sociocultural (e.g., family structure) and behavioral factors to predict depression risk in somatizing patients, particularly in East Asian contexts. It offers a practical tool for clinicians to prioritize high-risk individuals, reducing diagnostic delays and healthcare burdens. Future multicenter studies should validate its generalizability and incorporate biomarkers (e.g., inflammatory markers) to enhance mechanistic insights.

## Introduction

Somatization, a prevalent phenomenon in healthcare, refers to the presentation of physical symptoms that cannot be explained by known medical conditions or physiological abnormalities. These symptoms are often manifestations of underlying psychological distress, such as anxiety and depression. Common somatic complaints include palpitations, non-cardiac chest pain, dyspnea, gastrointestinal disturbances (e.g., irritable bowel syndrome, altered bowel habits), chronic fatigue, and diffuse musculoskeletal pain (1, 2). Globally, approximately 16–25% of individuals in primary care settings report somatic symptoms severe enough to meet criteria for somatization disorders, with higher prevalence in populations where cultural norms discourage direct expression of emotional distress (3). Patients exhibiting somatic symptoms frequently seek recurrent medical care, contributing to a significant healthcare burden. However, the inability to identify organic causes often complicates diagnosis and treatment, delaying appropriate psychological interventions (1, 4).

The relationship between Somatization and depression is well-documented, with studies confirming that individuals with depressive disorders are likely to experience somatic symptoms (5, 6). Beyond the epidemiological associations, there are complex pathophysiological mechanisms linking somatization symptoms and depression. At the neurotransmitter level, alterations in the serotonin and dopamine systems play crucial roles. Serotonin is involved in regulating mood, sleep, and pain perception. In patients with both somatization symptoms and depression, serotonin levels are often decreased, which can lead to mood disturbances and enhanced pain sensitivity, contributing to the manifestation of somatic symptoms. Dopamine, on the other hand, is related to motivation and reward. Imbalances in dopamine can cause anhedonia, a common symptom in depression, and may also be associated with the development of somatic complaints.

The hypothalamic - pituitary - adrenal (HPA) axis, a key component of the neuroendocrine system, is also dysregulated in these patients. Chronic stress, which is often associated with depression, can over - activate the HPA axis, leading to increased cortisol secretion. Elevated cortisol levels can have widespread effects on the body, including inflammation and immune system dysregulation. Inflammation has been linked to the development of somatic symptoms such as fatigue, muscle pain, and cognitive impairment, further blurring the line between physical and psychological symptoms. Furthermore, emerging evidence from neuroimaging studies shows structural and functional changes in the brains of patients with somatization symptoms and depression. Regions such as the prefrontal cortex, amygdala, and hippocampus, which are involved in emotion regulation, stress response, and memory, exhibit abnormal activity and connectivity. These changes may contribute to the complex interplay between psychological distress and the manifestation of somatic symptoms.

In China, cultural norms and expectations profoundly influence the manifestation and expression of psychological distress. Depression is frequently communicated through somatic complaints rather than direct emotional expressions, largely due to the persistent stigma surrounding mental illness. Patients often present to primary care physicians with persistent physical symptoms despite the absence of identifiable organic causes, a phenomenon known as cultural somatization (7, 8). This pattern has been extensively documented, revealing that Chinese patients are more likely to seek medical attention for physical ailments while minimizing or concealing emotional symptoms (1, 5). Empirical studies indicate that approximately 18.2% of outpatients in general hospitals report somatic complaints that meet diagnostic criteria for somatization disorders, highlighting the critical need for improved diagnostic frameworks in clinical practice (9).

Despite the well-documented association between somatic symptoms and depression, there remains a significant gap in research examining the predictive factors for depression among patients with somatization, both within China and globally (5, 6). Early identification and intervention are paramount, as untreated depression associated with somatic symptoms not only exacerbates the duration and severity of the illness but also increases the risk of adverse outcomes, including suicidal behavior (10).

In this context, the present study aims to develop a predictive risk model for depression in patients presenting with somatic symptoms. Guided by the methodological framework proposed by Collins et al. (11) for the development, validation, and updating of clinical prediction models, this research will identify independent predictors of depression among somatizing patients. By establishing a robust and clinically applicable risk assessment tool, this study seeks to facilitate early diagnosis, timely intervention, and improved clinical outcomes. The ultimate goal is to provide a personalized, evidence-based approach to risk stratification, enabling targeted treatment strategies, reducing unnecessary healthcare utilization, and enhancing long-term patient prognosis (10, 11).

## Object and method

### Research object

All patients enrolled were tracked throughout the 3, 6, and 12-month follow-up periods. No patients were lost to follow-up, ensuring the completeness of the dataset.

Population selection: Initially, a list of all patients admitted to the Department of General Medicine at xx Hospital from May 2020 to August 2022 was obtained. After excluding patients with incomplete medical records, those who had fully completed relevant examinations but still could not have their physical discomfort explained by known physiological or medical knowledge were identified. These patients were then evaluated using the Somatic Self Rating Scale (SSS) and PHQ-2. Only patients with SSS scores ≥ 38 (12) and PHQ - 2 scores<3 were included in the study. This step - by - step screening process ensured that the selected patients had significant somatization symptoms without current depressive symptoms, which was crucial for the development of the predictive model for depression risk.

Exclusion criteria: History of mental illness, paralysis, epilepsy, dementia, cognitive impairment, organ dysfunction, and other medical conditions.

This project uses SSS and PHQ-2 and subsequent PHQ-9 scales to evaluate Somatization symptoms and depressive status and conducts a self-made general questionnaire survey to screen patients with Somatization symptoms (SSS score ≥ 38) and non-depressive status (PHQ-2<3). Relevant factors are evaluated, and PHQ-2 questionnaires are used for 3-month, 6-month, and 12-month follow-ups to evaluate PHQ-2 and subsequent PHQ-9 scores and assess the patient’s depressive status.

### Evaluation tools

Life satisfaction: Measured using a 5-point Likert scale assessing general contentment with life. For ease of analysis, a score of 1-3 is considered dissatisfied, and 4-5 is considered satisfied. Self-health assessment: Patients rated their health on a scale from 1 (excellent) to 5 (worst). For ease of analysis, a score of 1-3 is considered poor, and 4-5 is considered good.

The Patient Health Questionnaire-2 (PHQ-2) was selected as a preliminary screening instrument due to its brevity and ease of administration, which minimizes patient burden in hospital settings. Although not a diagnostic tool, it is widely recognized for its validity as an initial screening measure for depressive symptoms.

The Somatic Symptom Scale (SSS) was employed to assess the somatic symptoms of patients (13). This scale, developed by Mao Jialiang from the Department of Cardiology at Renji Hospital in Shanghai (4th edition), demonstrates strong psychometric properties with a Cronbach’s α coefficient of 0.89 and a reliability coefficient of 0.96. The scale comprises 20 items, each rated on a 4-point Likert scale without reverse scoring. The scoring system is as follows: “1” indicates “no symptoms,” “2” indicates “mild degree,” “3” indicates “moderate degree,” and “4” indicates “severe degree.” Interpretation of SSS scores is as follows: scores <30 indicate no psychological or emotional issues requiring treatment; scores ≥30 and <38 suggest potential psychological and emotional problems, warranting psychological counseling; scores ≥38 and <42 indicate moderate psychological and emotional problems, recommending pharmacological treatment; and scores ≥42 signify severe psychological and emotional problems, necessitating combined pharmacological treatment (12).

The PHQ-2 and PHQ-9 instruments were utilized to screen patients for depressive disorders (9). The PHQ-2 scale consists of two items assessing the following symptoms over the past two weeks: (1) anhedonia or diminished interest in activities, and (2) depressed mood or feelings of hopelessness. Each item is scored on a 4-point scale: “not at all” (0 points), “several days” (1 point), “more than half the days” (2 points), and “nearly every day” (3 points). The total score ranges from 0 to 6, with a cutoff score of 3 indicating potential depression. Patients who screened positive on the PHQ-2 were subsequently administered the PHQ-9, which served as the diagnostic criterion for depression.

A self-administered general questionnaire was developed to collect comprehensive demographic and psychosocial information, including social demographics, marital status, family background, economic status, chronic medical conditions, social support systems, and life satisfaction indicators (9).

### Sample size and *post-hoc* power analysis

While a formal prospective power analysis was not conducted due to the exploratory nature of this prediction model study, we ensured methodological rigor through alternative approaches: Sample Size Justification: Based on recommendations for prediction models requiring at least 10 events per predictor variable (EPV), our model included 5 predictors with 34 depression events (6.8 EPV), meeting the minimum threshold for stable estimation. *Post-Hoc* Power Analysis: Using the pROC package in R, a *post-hoc* power calculation based on the observed AUC (0.81) and sample size (n=200) indicated >80% power to detect an AUC significantly greater than 0.70 (null hypothesis) at α=0.05. Internal Validation: Bootstrap resampling (200 iterations) yielded a narrow optimism-corrected AUC confidence interval (0.728–0.893), confirming robust discrimination despite the moderate sample size.

### Statistical methods

Statistical analyses were performed using SPSS 26.0 and R 4.2 software. Continuous data that did not follow a normal distribution were expressed as medians with interquartile ranges (IQR). Group comparisons were conducted using independent two-sample t-tests.

Variable selection was performed using LASSO (Least Absolute Shrinkage and Selection Operator) regression, followed by the development of a predictive model through multivariate logistic regression analysis, which was subsequently visualized using a nomogram. Prior to LASSO regression, variables exhibiting a variance inflation factor (VIF) exceeding 5 were comprehensively evaluated to address multicollinearity. To further mitigate overfitting, a 10-fold cross-validation approach was employed during LASSO regression to optimize the penalty parameter (λ). Internal validation of the final logistic regression model was conducted using 200 bootstrap resamples to calculate optimism-adjusted performance metrics.

The predictive model was evaluated based on three key aspects: discrimination, calibration, and clinical utility. Discrimination was assessed using the area under the receiver operating characteristic curve (ROC-AUC). Calibration was evaluated using calibration curves and the Hosmer-Lemeshow goodness-of-fit test. Clinical utility was examined using Decision Curve Analysis (DCA) to determine the net benefit across different threshold probabilities. A p-value of less than 0.05 was considered statistically significant.

### Ethical approval

This study was conducted in accordance with the tenets of the Declaration of Helsinki. The procedures adopted were in accordance with the ethical standards formulated by the Ethics Committee of Longyan First Hospital, ethics approval number LYREC2022-006-01. Written informed consent will be obtained from all participants or their legal guardians before the study.

## Results

### Basic characteristics of the study cohort

The study included 200 patients aged between 30 and 97 years, with a mean age of 67.77 ± 12.11 years. The cohort consisted of 110 males (55%) and 90 females (45%). Among the participants, 34 patients (17%) were diagnosed with depression. The mean Somatic Symptom Scale (SSS) score was 34.85 ± 8.31. Detailed demographic and clinical characteristics of the enrolled patients are presented in **Table 1**.

**Table 1:** Comparison of basic characteristics of patients with somatization symptoms.

Characteristic	Depression		p-value
	No, N = 166^1^	Yes, N = 34^1^	
**Age [Median (IQR)]**	68 (60, 75)	77 (68, 82)	<0.001^2^
**Gender**			0.520^3^
Female	93 (56.0%)	17 (50.0%)	
Male	73 (44.0%)	17 (50.0%)	
**Education**			0.051^3^
High school	71 (42.8%)	11 (32.4%)	
Primary school and below	67 (40.4%)	21 (61.8%)	
Undergraduate	28 (16.9%)	2 (5.9%)	
**Employment**			0.089^4^
Employee	22 (13.3%)	1 (2.9%)	
Farmer	54 (32.5%)	8 (23.5%)	
Retire	44 (26.5%)	9 (26.5%)	
Unemployed	46 (27.7%)	16 (47.1%)	
**Marriage**			0.180^4^
Married	146 (88.0%)	27 (79.4%)	
Unmarried/separation/spouse	20 (12.0%)	7 (20.6%)	
**Income**			<0.001^3^
50000 to 100000	47 (28.3%)	4 (11.8%)	
Less than 50000	33 (19.9%)	18 (52.9%)	
More than 100000	86 (51.8%)	12 (35.3%)	
**Life satisfaction**			0.211^3^
Dissatisfied	93 (56.0%)	23 (67.6%)	
Satisfied	73 (44.0%)	11 (32.4%)	
**Self-Health Assessment**			0.057^3^
Good	140 (84.3%)	24 (70.6%)	
Poor	26 (15.7%)	10 (29.4%)	
**Smoking**			0.531^3^
No	113 (68.1%)	25 (73.5%)	
Yes	53 (31.9%)	9 (26.5%)	
**Drinking**			0.535^3^
No	121 (72.9%)	23 (67.6%)	
Yes	45 (27.1%)	11 (32.4%)	
**Somatic pain**			0.488^3^
None	36 (21.7%)	5 (14.7%)	
Slight	90 (54.2%)	18 (52.9%)	
Medium to severe	40 (24.1%)	11 (32.4%)	
**Traditional Chinese Medicine in the past month**			0.430^3^
No	66 (39.8%)	16 (47.1%)	
Yes	100 (60.2%)	18 (52.9%)	
**Social interaction in the past month**			0.010^3^
No	77 (46.4%)	24 (70.6%)	
Yes	89 (53.6%)	10 (29.4%)	
**Number of chronic diseases**			0.556^4^
0	41 (24.7%)	6 (17.6%)	
1	61 (36.7%)	11 (32.4%)	
2	39 (23.5%)	9 (26.5%)	
≥3	25 (15.0%)	8 (23.5%)	
**Number of children**			0.130^4^
1	47 (28.3%)	4 (11.8%)	
2	62 (37.3%)	16 (47.1%)	
≥3	57 (34.3%)	14 (41.2%)	
**Living with children**			<0.001^3^
No	41 (24.7%)	20 (58.8%)	
Yes	125 (75.3%)	14 (41.2%)	
**Self medication**			0.001^3^
No	71 (42.8%)	25 (73.5%)	
Yes	95 (57.2%)	9 (26.5%)	
**Deterioration of the memory**			0.083^3^
No	100 (60.2%)	15 (44.1%)	
Yes	66 (39.8%)	19 (55.9%)	

^1^n (%).

^2^Wilcoxon rank sum test.

^3^Pearson's Chi-squared test.

^4^Fisher's exact test.

### Variable selection using LASSO regression

**Figure 1:**
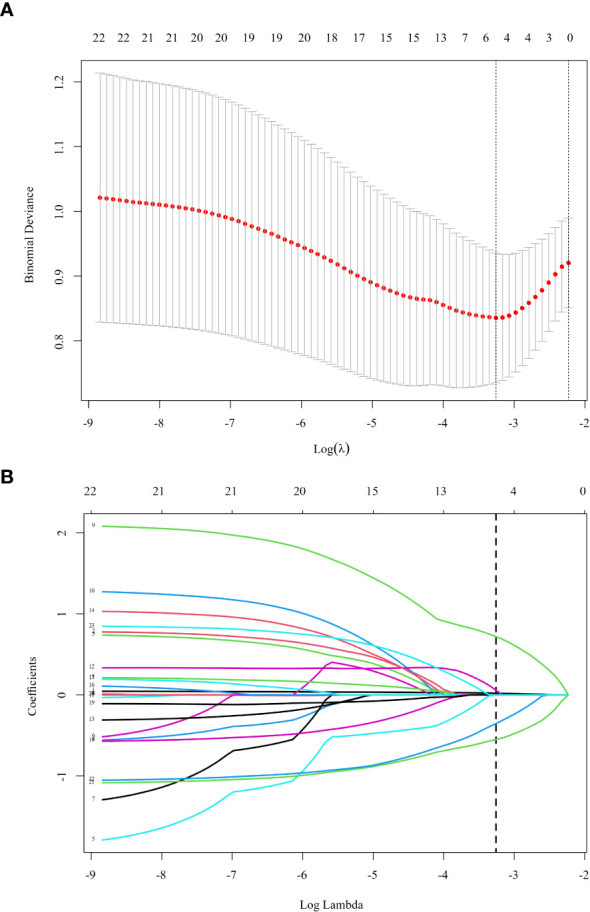
Using the LASSO regression model to screen predictive variables. **(A)** is the LASSO coefficient curve for 19 variables, and **(B)** is the process of screening λ through 10-fold cross-validation in the LASSO model. LASSO regression helps reduce the risk of overfitting by penalizing complex models and selecting only the most predictive variables for depression risk.

**Figure 2:**
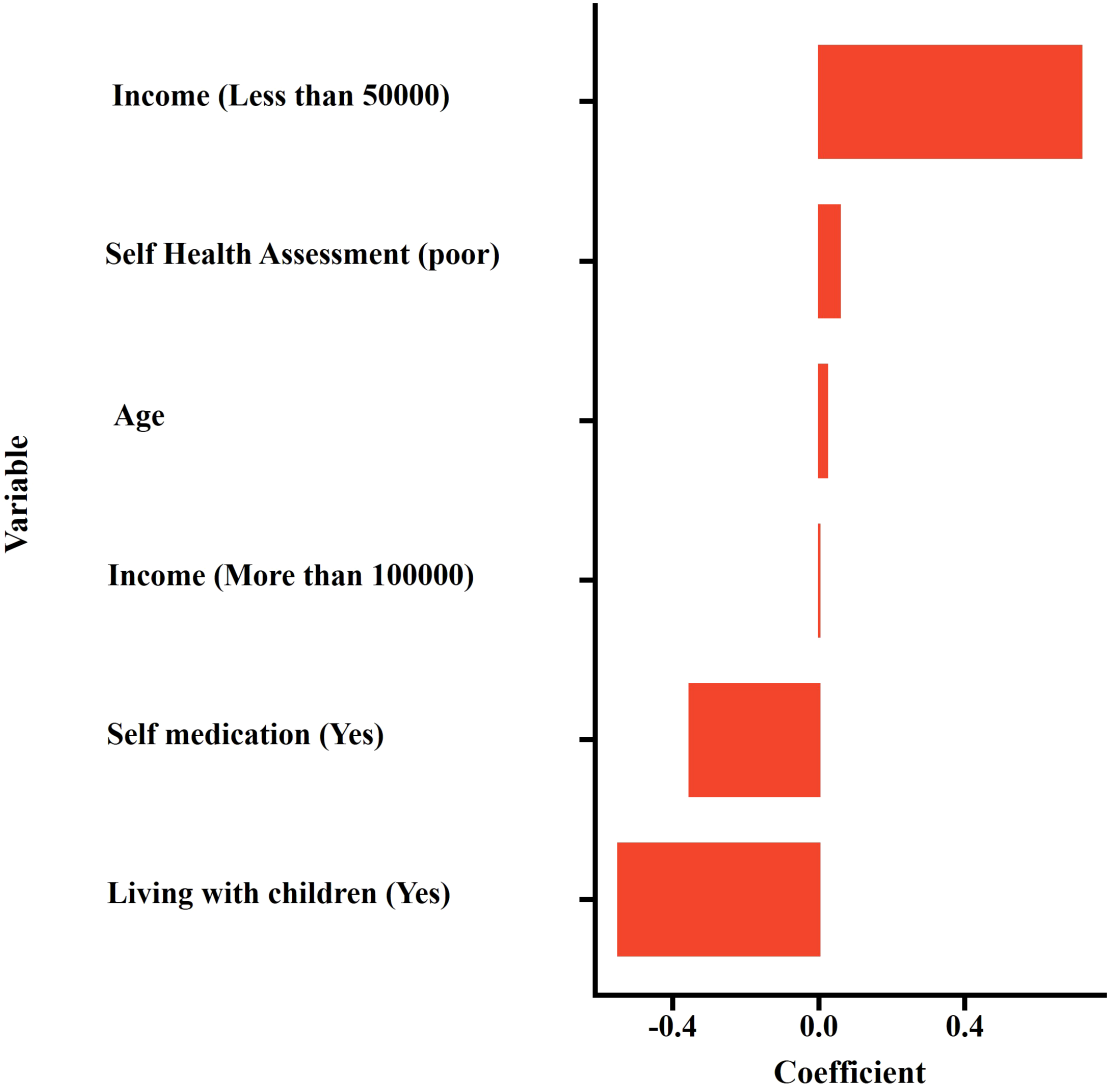
Lasso regression coefficients of filtered variables.

LASSO regression analysis was employed to screen predictive variables from 18 candidate variables, including general social information, family status, economic status, chronic disease history, and life satisfaction. At the optimal penalty parameter (λ = 0.038), five non-zero coefficient predictive variables were identified (**Figures 1**, **2**). The selected variables included age, income, self-rated health status, cohabitation with children, and self-medication practices. The LASSO regression model demonstrated optimal performance at this λ value.

### Development of the predictive model

**Figure 3:**
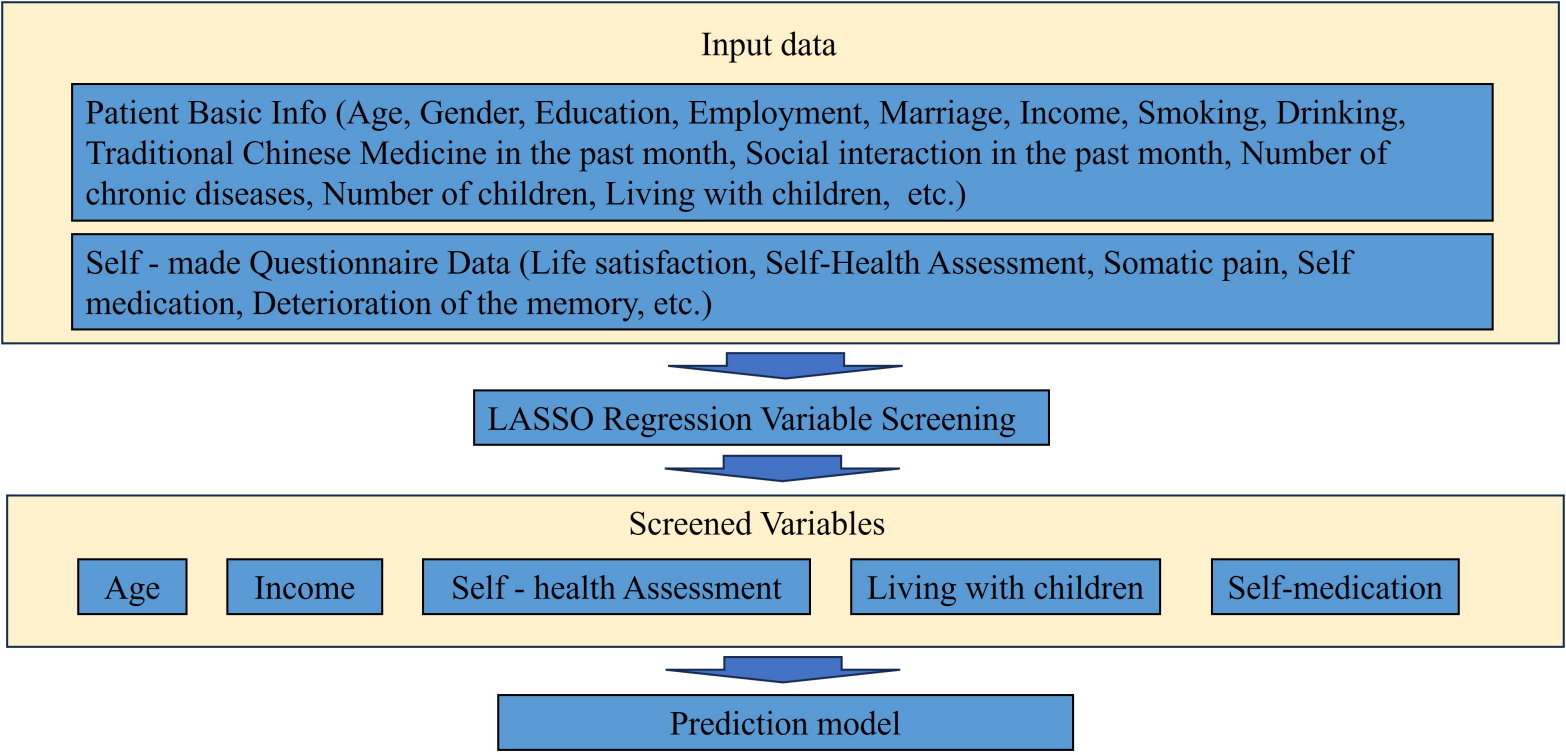
Variable filtering flow chart.

A multivariate logistic regression model was constructed to predict depression in patients with somatization symptoms. The dependent variable was the presence of depression, while the independent variables were the five predictors identified through LASSO regression analysis (**Figure 3**). The results indicated that self-rated health status, age, self-medication practices, and cohabitation with children were significant risk factors for depression in patients with somatization symptoms (p < 0.05), as detailed in **Table 2**.

**Table 2:** Multivariate analysis of depression in patients with somatization symptoms.

Characteristic	Univariable					Multivariable				
	N	Event N	OR^1^	95% CI^1^	p-value	N	Event N	OR^1^	95% CI^1^	p-value
**Age**	200	34	1.07	1.03, 1.12	<0.001	200	34	1.06	1.01, 1.11	0.018
Income										
50000 to 100000	51	4	—	—		51	4	—	—	
Less than 50000	51	18	6.41	1.99, 20.68	0.002	51	18	6.46	1.84, 22.68	0.004
More than 100000	98	12	1.64	0.50, 5.37	0.414	98	12	3.23	0.87, 11.97	0.079
Self-Health Assessment										
poor	36	10	—	—		36	10	—	—	
good	164	24	0.45	0.19, 1.04	0.062	164	24	0.47	0.18, 1.24	0.127
Living with children										
No	61	20	—	—		61	20	—	—	
Yes	139	14	0.23	0.11, 0.50	<0.001	139	14	0.41	0.17, 0.99	0.047
Self-medication										
No	96	25	—	—		96	25	—	—	
Yes	104	9	0.27	0.12, 0.61	0.002	104	9	0.42	0.16, 1.07	0.069

^1^OR, Odds Ratio; CI, Confidence Interval; AIC, 157; R^2^, 0.301

**Figure 4:**
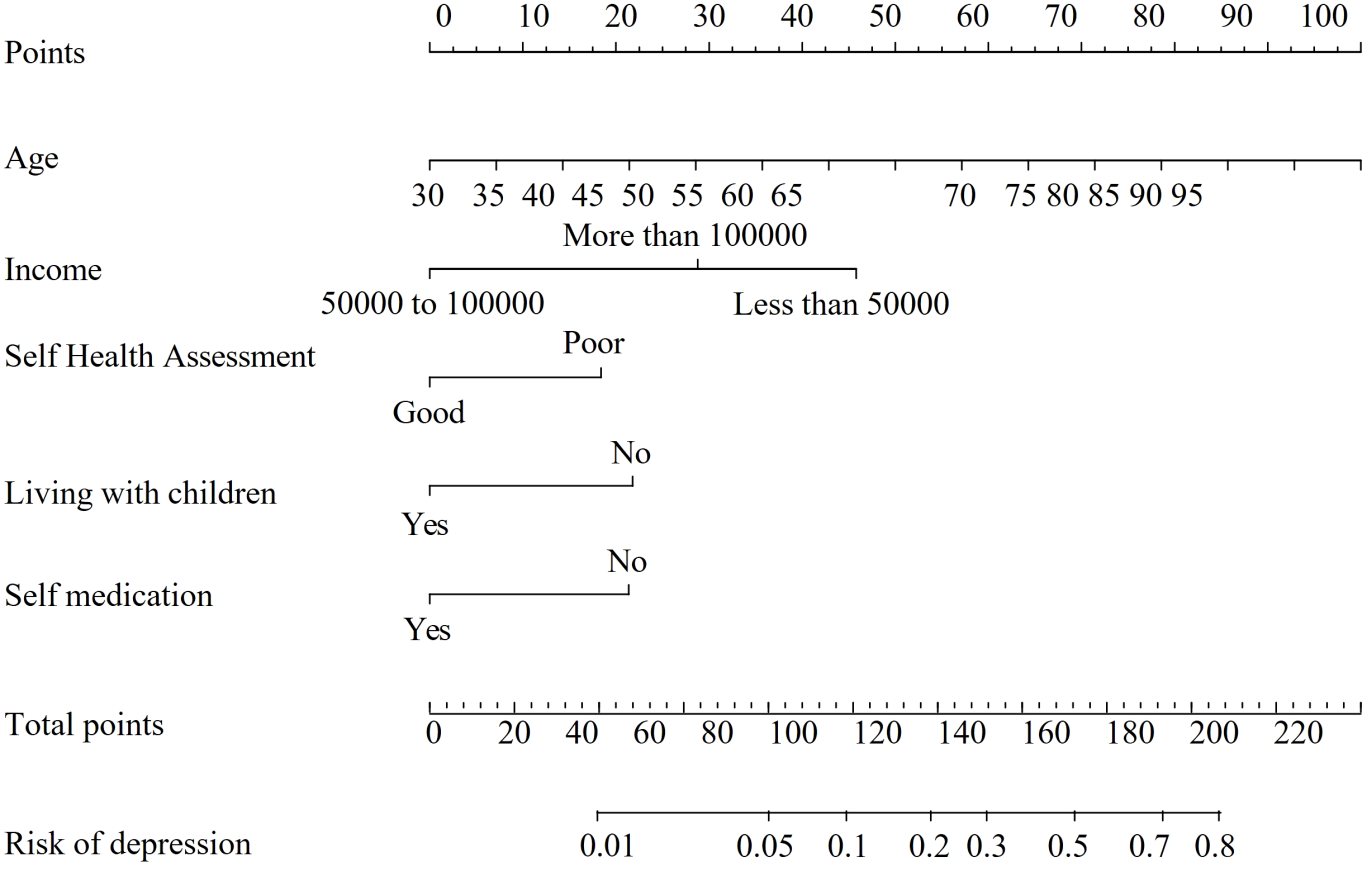
ROC curve of the predictive model for predicting depression in patients with somatization. Bootstrap resampling was performed 200 times, yielding an AUC of 0.795 (95% CI: 0.718-0.88). This curve demonstrates the trade-off between sensitivity and specificity, with higher AUC values indicating better model performance.

A nomogram was developed based on the predictive variables to facilitate clinical application (**Figure 4**). The nomogram allows clinicians to assign scores to each variable based on patient-specific values, sum the scores, and determine the corresponding risk of depression by drawing a vertical line from the total score axis.

### Instructions for using the momogram

1. Locate the patient’s value for each predictor variable on the corresponding axis.
2. Draw a vertical line to the points axis to determine the score for each variable.
3. Sum the scores for all predictors.
4. Draw a vertical line from the total score axis to the risk axis to estimate the probability of depression.

We then performed subgroup analyses to explore whether there was an interaction in the inclusion measures. In the subgroup analysis, there was no significant interaction between income level and age and depression risk (P for interaction = 0.272). Specifically, age was significantly associated with depression risk in people with incomes below $50,000 (OR = 1.11, 95% CI: 1.02-1 20, P = 0.015), but not in people with incomes between $50,000 and $100,000 and above $100,000 (P = 0.064 and 0.221, respectively). There was no significant interaction between age and depression risk in self-assessed health (P for interaction = 0.809), and age was significantly associated with depression risk in the self-assessed healthy population (OR = 1.07, 95% CI: 1.03-1, P = 0.002), but not in the self-assessed healthy population (P = 0.076). Self-medication did not have a significant interaction with age and depression risk (P for interaction = 0.144), and age was significantly associated with depression risk in the non-self-medication population (OR = 1.09, 95% CI: 1.03-1, P = 0.002), but not in the self-medication population (P = 0.312). Living with children did not have a significant interaction between age and depression risk (P for interaction = 0.596). Among those living without children, age was significantly associated with depression risk (OR = 1.08, 95% CI: 1.01-1, P = 0.028), while among those living with children, the association was nearly significant (P = 0.053). The results of the subgroup analysis are shown in **Table 3**.

**Table 3:** Subgroup analysis if Univariate Logistic Model.

Subgroup	N	Crude OR (95% CI)	P value	P for interaction
Overall	200	1.07 (1.03-1.12)	<0.001	
Income				0.272
50000 to 100000	51	1.12 (0.99-1.27)	0.064	
Less than 50000	51	1.11 (1.02-1.20)	0.015	
More than 100000	98	1.04 (0.98-1.10)	0.221	
Self-Health Assessment				0.809
good	164	1.07 (1.03-1.12)	0.002	
poor	36	1.09 (0.99-1.19)	0.076	
Self-medication				0.144
No	96	1.09 (1.03-1.16)	0.002	
Yes	104	1.03 (0.97-1.09)	0.312	
Living with children				0.596
No	61	1.08 (1.01-1.15)	0.028	
Yes	139	1.05 (1.00-1.11)	0.053	

### Analysis of the rationality of prediction models

**Figure 5:**
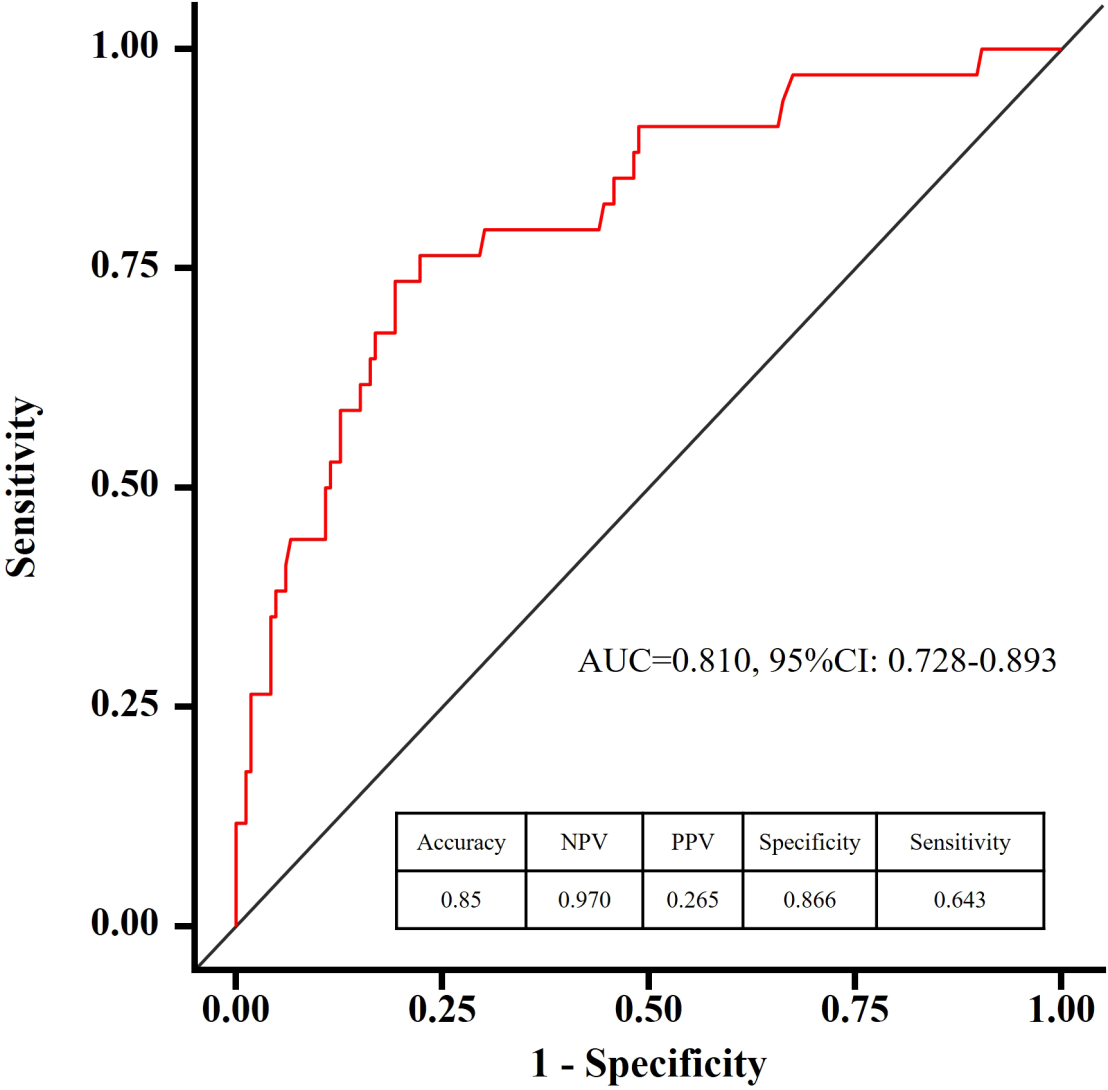
Calibration curve of the prediction model. Bootstrap resampling was performed 1000 times to validate calibration. This curve compares predicted versus observed probabilities. A model with perfect calibration will have predictions close to the diagonal line, indicating good agreement between predicted and actual risks.

**Figure 6:**
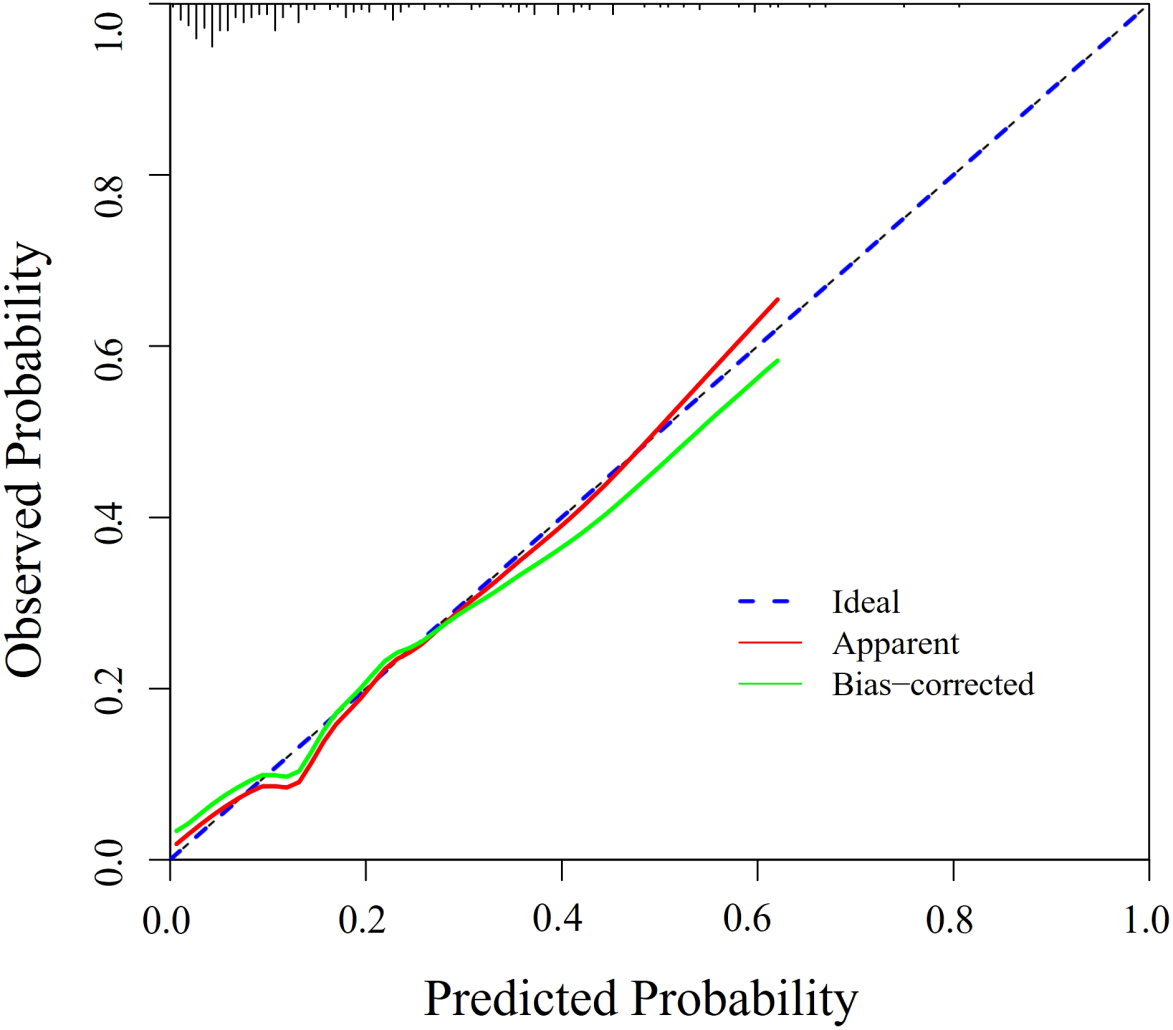
DCA analysis of the prediction model. Bootstrap resampling was performed 1000 times with 10-fold cross-validation. Decision Curve Analysis (DCA) illustrates the net clinical benefit of applying the model at different threshold probabilities. A higher net benefit across a range of thresholds indicates the model’s utility in clinical decision-making.

Discrimination and calibration of the predictive model are the primary means of validation. Discrimination of the model is assessed by plotting ROC curves for the prediction of depressive states in patients with Somatization symptoms. The sample size of this study is 200 cases, not grouped, and all data are used as a modeling queue. The AUC of the predictive model is 0.810 (95% CI: 0.728 - 0.893), indicating good discrimination of the model (**Figure 5**). In this series, there was good consistency in the calibration curve of the depression risk nomogram for patients with Somatization symptoms (**Figure 6**).

These findings indicate that the model demonstrates reasonable goodness of fit, with predicted probabilities closely aligning with observed probabilities, suggesting excellent calibration. In summary, the prediction model exhibits moderate predictive capability.

**Figure 7:**
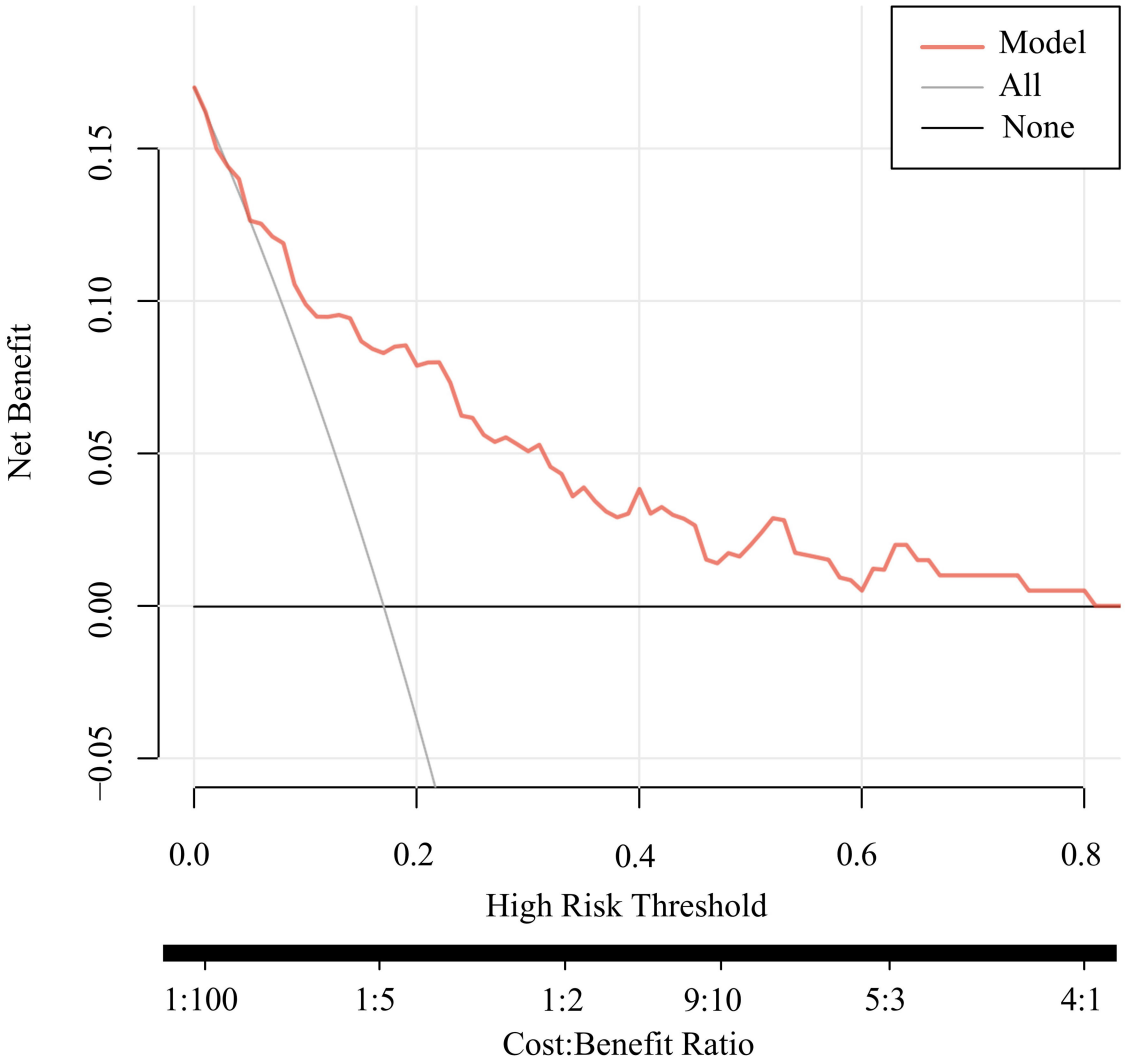
Line chart of the predictive model for the risk of depression in patients with Somatization symptoms. Self-health evaluation: 1 (good), 2 (average), 3 (poor); Living with children: 1 (Yes), 0 (No); Self-medication: 1 (Yes), 0 (No). To use the nomogram, identify the patient’s values on each predictor axis. Draw a vertical line to the points axis to assign a score. Sum the scores for all predictors and draw a vertical line from the total score axis to determine the corresponding risk of depression.

**Figure 7** presents the decision curve analysis (DCA) for assessing the risk of depression in patients with somatization symptoms. DCA is a clinically relevant method for evaluating the net benefit and clinical utility of predictive models. The analysis revealed that when the threshold probabilities for both patients and clinicians exceed 5%, using this nomogram to predict depression risk provides greater clinical benefit compared to implementing intervention plans for all patients. Within this threshold range, the net benefit of the prediction model significantly surpasses that of the two extreme scenarios (intervening for all patients or no patients). This underscores the model’s potential to guide targeted clinical decision-making, optimizing resource allocation and improving patient outcomes.

## Discussion

The study findings reveal that cohabitation with children and positive self-rated health evaluations serve as protective factors against depression, consistent with intergenerational studies highlighting the role of family support in mental health (14). Additionally, age-related physical decline significantly increases vulnerability to depression (15). These results underscore the importance of addressing both physical and social determinants to mitigate depression risk in patients with somatic symptoms (14–17).

Nomograms are widely recognized as a reliable and practical tool in clinical research for disease prediction, particularly in oncology and chronic disease management (18, 19). By analyzing risk factors associated with disease onset, progression, and prognosis, nomograms provide an intuitive and user-friendly interface for predicting disease probability, aiding clinicians in making informed decisions (20, 21).

In the developmental cohort of this study, 34 patients (17%) with somatic symptoms were diagnosed with depression. LASSO regression identified seven independent risk factors for depression, including self-rated health, memory decline, age, recent social status, annual family income, self-medication practices, and cohabitation with children. Multivariate logistic regression analysis confirmed that self-rated health, age, self-medication, and cohabitation with children were significant predictors of depression in patients with somatization symptoms (p < 0.05). The bootstrap-corrected area under the curve (AUC) for the nomogram in the training set was 0.810 (95% CI: 0.728–0.893), indicating strong discriminatory power. Furthermore, the Hosmer-Lemeshow goodness-of-fit test demonstrated excellent calibration, with predicted risks closely aligning with observed risks.

This predictive model highlights that improving self-rated health, fostering cohabitation with children, and addressing self-medication practices can reduce depression risk in patients with somatization symptoms, while advancing age exacerbates this risk. These findings align with prior studies in primary care and outpatient settings (22–24).

Existing literature suggests that aging increases depression risk due to factors such as multimorbidity, declining physical and cognitive function, and reduced socioeconomic status (16). Our study corroborates that older individuals with somatic symptoms face a higher risk of depression, likely due to physical frailty, increased dependency, social isolation, and financial insecurity (25–27). As China undergoes rapid population aging, the burden of depression among the elderly is expected to rise. Proactive prevention and intervention strategies are urgently needed to address this growing public health challenge.

Our study found that patients with somatic symptoms with low self-health evaluations had higher risk of developing depression compared to patients with high self-health evaluations. A study found that poor self-health assessment is a risk factor for developing depressive states in individuals aged 65 and above (28). As age increases, physiological functions gradually decline, self-care abilities decrease, and dependence increases, all of which may lead to poor self-health evaluations in the elderly. These factors have been reported as risk factors for the onset of depression in the elderly in other studies (16). When daily activities are restricted due to joint pain and prolonged sitting, physical function is impaired, and activity is restricted, they have a lower evaluation of their health, which is related to their occurrence of depression (29).

Research indicates that housing type does not directly influence the occurrence of depressive symptoms in older adults, whereas intergenerational support serves as a protective factor against depression (14). This suggests that while family living arrangements alone may not significantly impact depression in the elderly, enhancing the level of intergenerational support and improving older adults’ perceptions of such support are critical (14). The findings of this study further demonstrate that cohabitation with children is a protective factor against depression in patients with somatization symptoms. This may be attributed to the cultural context of the study population. In the Longyan region, a significant proportion of the population resides in rural and township areas, where multigenerational households are common. Studies have shown that such living arrangements facilitate greater emotional communication between older adults and their children or grandchildren, providing stronger family support (24). Higher levels of family support are associated with a lower incidence of depressive symptoms among older adults in China (30).

Patients often prioritize reporting physical symptoms over psychological concerns when consulting non-psychiatrists, as somatic symptoms can obscure the core manifestations of depression, thereby delaying its diagnosis (10, 31, 32). Our findings highlight the importance of alerting non-psychiatrists to the potential presence of mental health issues in patients presenting with somatization symptoms. Specifically, patients with somatization symptoms who report poor self-rated health, are older, lack self-treatment capabilities, and do not live with their children are at higher risk of depression. It is crucial to recognize that the severity of somatic symptoms does not necessarily reflect the severity of the underlying disease.

Existing research has documented the efficacy of pharmacological interventions (33) and psychological therapies (25, 26, 34, 35) for somatization symptoms. Accurate identification of these patients can significantly alleviate individual suffering and reduce the broader societal burden of disease. Clinicians should therefore adopt a holistic approach, integrating both physical and psychological assessments, to ensure timely and effective interventions for this vulnerable population.

The predictive model in this study primarily identified the contribution of sociobehavioral factors (e.g., self-health assessments, living with children) to depression risk, but acknowledged that the physiological basis of somatization symptoms (e.g., immune activation, neuroendocrine disorders) may indirectly affect outcomes through unmeasured pathways. For example, chronic inflammatory states may simultaneously cause physical discomfort (e.g., joint pain, fatigue) and low mood (36), while social support (e.g., living with children) may buffer such physiological risks by modulating stress responses (e.g., lowering cortisol levels) (37). Future studies should combine biomarkers (e.g. IL-6, CRP) and psychosocial variables to uncover the multilevel mechanisms underlying the somatization-depression relationship. Future research needs to obtain more comprehensive biopsychosocial data through multicenter collaboration to validate and extend the explanatory power of this model.

In clinical applications, it is important to note that, first, the protective effect of “living with children” may be closely related to traditional Chinese family values (e.g., filial piety, intergenerational support) (14); second, somatization as an expression of psychological symptoms is more prevalent in East Asian cultures (10), while Western patients may report emotional symptoms more directly. Therefore, caution is required when applying this model directly to Western populations, and it is recommended to adjust variable definitions (e.g., replacing “living with children” with “social support network density”) and perform cross-cultural calibration.

It should be noted that this study has certain limitations. The data used were from a single hospital in China, which may lead to bias in patient characteristics due to regional differences, different medical service levels, and unique cultural backgrounds. As a result, the generalizability of our findings may be limited. Future studies could consider a multi - center design to overcome these limitations and obtain more comprehensive and representative results. This would enhance the applicability of the predictive model in diverse healthcare settings. Another major limitation of this study is related to the assessment of subliminal depressive status evaluated by PHQ - 2 and subsequent PHQ-9. The MINI (Mini-International Neuropsychiatric Interview), was better, but it is not used because it is not convenient enough.

## Conclusion

This study developed a personalized risk prediction model incorporating four key variables: self-rated health, age, self-treatment practices, and cohabitation with children. The model demonstrates robust predictive performance and clinical utility, as evidenced by its ROC curve, calibration curve, and decision curve analysis (DCA). By leveraging these factors, healthcare providers can make evidence-based decisions to identify and mitigate depression risk in patients with somatic symptoms. This approach not only enhances patient outcomes but also alleviates the individual and systemic burden on healthcare resources.

## Data Availability

The raw data supporting the conclusions of this article will be made available by the authors, without undue reservation.
